# Corticospinal Excitability Preceding the Grasping of Emotion-Laden Stimuli

**DOI:** 10.1371/journal.pone.0094824

**Published:** 2014-04-14

**Authors:** Anaelli Aparecida Nogueira-Campos, Laura Alice Santos de Oliveira, Valeria Della-Maggiore, Paula Oliveira Esteves, Erika de Carvalho Rodrigues, Claudia D. Vargas

**Affiliations:** 1 Laboratory of Neurobiology II, Neurobiology Program, Institute of Biophysics Carlos Chagas Filho, Federal University of Rio de Janeiro, Rio de Janeiro, Brazil; 2 Department of Physiology, Federal University of Juiz de Fora, Juiz de Fora, Brazil; 3 Post-graduate Program in Rehabilitation Sciences, Augusto Motta University Center, Rio de Janeiro, Brazil; 4 Department of Physiology and Biophysics, School of Medicine, University of Buenos Aires, Buenos Aires, Argentina; University of Ottawa, Canada

## Abstract

Evolutionary theories posit that emotions prime organisms for action. This study examined whether corticospinal excitability (CSE) is modulated by the emotional valence of a to-be-grasped stimulus. CSE was estimated based on the amplitude of motor evoked potentials (MEPs) elicited using transcranial magnetic stimulation (TMS) and recorded on the *first dorsal interosseous* (FDI) muscle. Participants were instructed to grasp (ACTION condition) or just look at (NO-ACTION condition) unpleasant, pleasant and neutral stimuli. TMS pulses were applied randomly at 500 or 250 ms before a go signal. MEP amplitudes were normalized within condition by computing a ratio for the emotion-laden stimuli by reference to the neutral stimuli. A divergent valence effect was observed in the ACTION condition, where the CSE ratio was higher during the preparation to grasp unpleasant compared to pleasant stimuli. In addition, the CSE ratio was lower for pleasant stimuli during the ACTION condition compared to the NO-ACTION condition. Altogether, these results indicate that motor preparation is selectively modulated by the valence of the stimulus to be grasped. The lower CSE for pleasant stimuli may result from the need to refrain from executing an imminent action.

## Introduction

Emotions prime organisms for actions. In humans, the influence of emotions upon the motor system has been investigated by presenting pictures and videos depicting emotional contexts while measuring motor outcomes. For example, the viewing of emotional-laden pictures enhances spinal [1], [2] and startle reflexes [3]–[6], modulates postural control [7], [8], affects reaction times [9]–[12], influences force production [13], and affects arm/eye kinematic profiles [14], [15].

The effect of emotional contexts upon neural activity in motor-related areas has also been examined [16]–[20]. Employing fMRI, Pereira et al (2010) showed increased activity in motor-related areas when a simple reaction time task was performed after viewing unpleasant compared to neutral pictures. By recording the readiness potential, an electrophysiological marker of motor preparation, Grecucci et al. (2009) described higher brain activity after viewing unpleasant compared to neutral pictures. Using a similar approach, de Oliveira et al. (2012) showed that the readiness potential was larger preceding the grasping of unpleasant and reduced preceding the grasping of pleasant as compared to neutral stimuli. In addition, a stronger inhibitory activity was described for positive contexts when event-related potentials were recorded in a go/no-go paradigm. Interestingly, this observation came out when participants had to withhold an imminent response [19], [20]. Taken together, these results indicated that motor-related areas are susceptible to the action's emotional content.

The effects of emotion on action have also been investigated using transcranial magnetic stimulation (TMS) [21]–[28]. This technique is recognized as a key tool for assessing changes in corticospinal excitability (CSE) [29]–[31]. Results from TMS studies were consistent with a CSE modulation based on arousal when emotional pictures were either viewed passively [21]–[23], [25] or associated with movement preparation [24], [28]. Consistent with the idea that emotion induces action predispositions [4], [32], it is reasonable to suppose that preparing to act upon an emotion-laden stimulus may be affected by the emotional content of the stimulus. Although some evidence supports this proposal [17], [18], [24], [28], the effects of emotion on CSE during motor preparation when an individual is required to interact with the source of the emotion remain largely unexplored.

Motor preparation results from changes in the motor systems that prime the organism for efficient interactions with the environment [33], [34]. To select an appropriate action, the individual must assess the context in which the action will occur as well as the properties of the objects with which he/she will interact [35]–[37]. Convergent experimental evidence indicates that motor programs selected during motor preparation are shaped by the action's goal [38]–[42]. In the present study, we tested the hypothesis that motor preparation is modulated by valence when the emotion is inherent to the target of the action. For this purpose, we measured CSE while participants prepared to grasp a set of emotion-laden stimuli that differed in terms of valence but yielded similar levels of arousal. We hypothesized that CSE preceding a grasping movement would vary depending on the valence of the stimulus to be grasped. More specifically, we predicted that CSE would decrease for pleasant stimuli, reflecting the mechanisms needed to refrain from executing an imminent action, whereas CSE would increase for unpleasant stimuli, reflecting the need to overpass brain networks recruited by the unwillingness to act.

## Materials and Methods

### Ethics Statement

All participants provided informed written consent for their participation in the study, which was approved by the ethics committee of the Clementino Fraga Filho University Hospital at the Federal University of Rio de Janeiro (004/09) and conducted according to the Declaration of Helsinki.

### Participants

Fourteen right-handed male undergraduate and graduate students between the ages of 21 and 36 years (mean 27.7, S.D. 4.12) participated in this study. Participants had no personal or family history of epilepsy and did not present with any neurological or psychiatric disorders. Handedness was assessed through the Edinburgh Inventory [43].

### Stimulus Selection

A set of 60 stimuli, consisting of transparent cylinders sealed with plastic film containing emotion-laden objects, was categorized by means of the *Self-assessment Manikinscale* (SAM), as in a previous study [18]. This is an affective rating scale in which each stimulus is classified in the valence and arousal dimensions [44]. Using this scale, participants classified their interactions with each stimulus in both dimensions. In this system, the ratings of valence are indicated by graphical representations of facial expressions ranging from a severe frown (most negative) to a broad smile (most positive). For arousal, this scale varies from a state of low to high agitation. Participants may select any of the five figures, or the spaces in between, on a nine-point rating scale for each dimension. In the valence dimension, 9 represent the extreme of pleasantness, and 1 represents the extreme of unpleasantness. Likewise, for arousal, 9 represent a high rating, and 1 represents a low rating. From the set of 60 rated stimuli, a total of 42^1^ stimuli, i.e., 14 from each emotional category were selected. The 14 stimuli whose valence was rated between 2.0 and 4.0 were classified as unpleasant, the 14 stimuli rated between 6.5 and 8.5 were classified as pleasant, and the 14 stimuli rated between 4.5 and 5.5 were classified as neutral. The unpleasant and pleasant stimuli had similar levels of arousal, and both had higher levels of arousal compared to the neutral stimuli.

To equate the hand aperture of participants during the action, identical cylinders (height: 9.7 cm and radius: 3.5 cm) were employed. Furthermore, stimulus weights were balanced across emotional categories (pleasant: 312.5±38.9 g; unpleasant: 314.3±38.9 g; and neutral: 292.9±48.5 g). A one-way ANOVA yielded no statistically significant differences in weight [F (2,39)  = 1.10, p = 0.34].

^1^NOTE: Selected stimuli: PLEASANT: Chocolate, money, wrapped condom, mobile phone, soccer cards, toys, gold trophy, ball, candy, television remote control, MP3 player, marbles, wrist watch and pocket game; UNPLEASANT: cake with hair, embalmed vomit, embalmed cockroach, artificial excrement, embalmed gizzard, rotten food, bluebottle fly on a biscuit, embalmed dead rat, rotten artichoke, embalmed chicken foot, artificial spider, artificial snake, embalmed fish eye and dentures; NEUTRAL: eraser, adhesive tape, pencil sharpener, crumpled paper ball, silver paper clips, binder clips, sponge, stick glue, plastic bag, alkaline battery, cotton balls, pieces of colored wire, spun wool and strip of staples.

### Procedure

**Figure 1:**
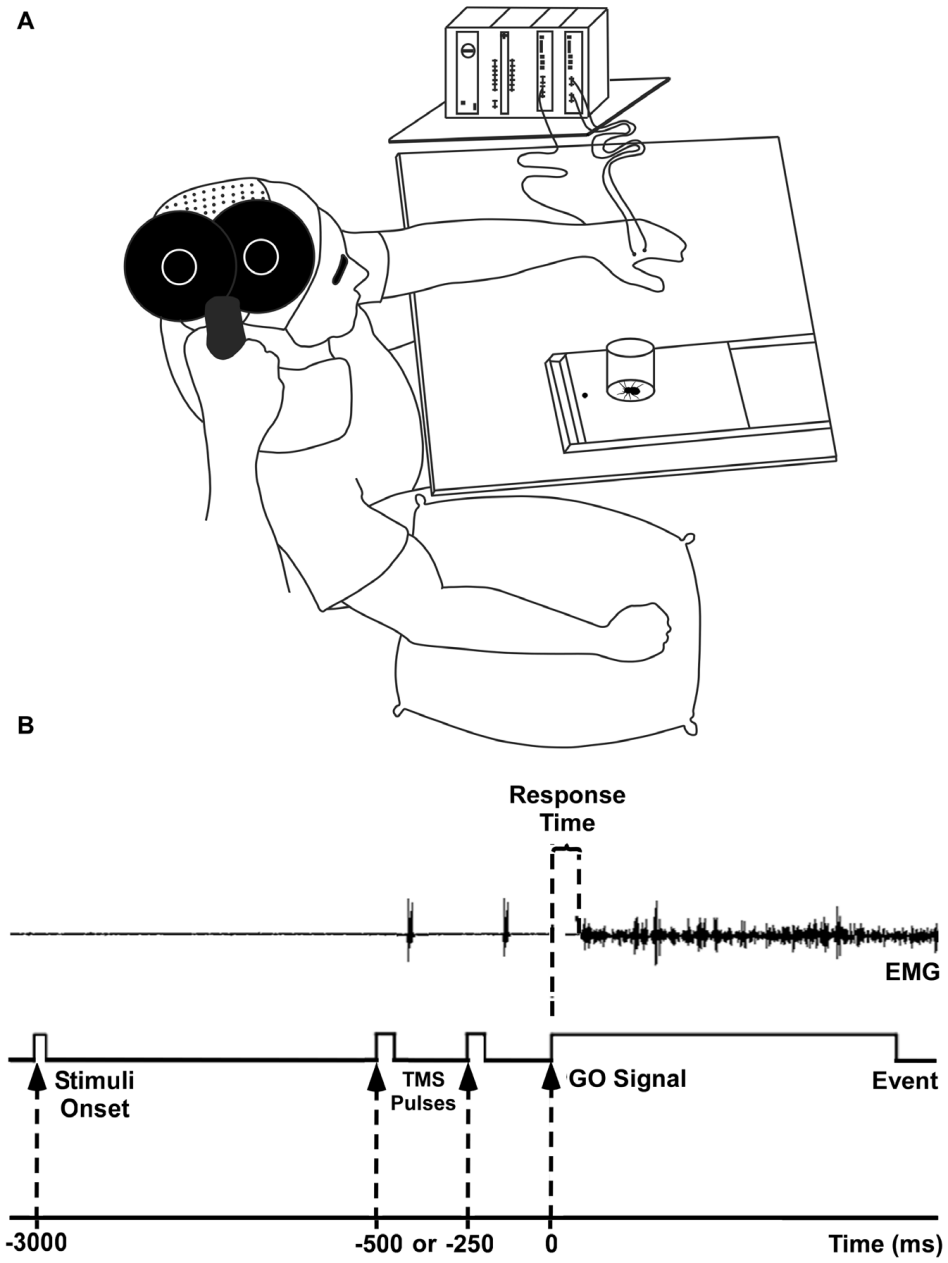
Experimental design. A) The participant sat behind a table with his left arm placed over the table. The right arm rested on a pillow throughout the experimental session. The TMS coil was positioned over the motor cortex by an experimenter. Each stimulus (cylinder containing the emotion-laden object) was presented separately on a sliding slab. Upon presentation, the participants were instructed to wait for a red light to turn on. They then had to grasp or just look at the stimulus. EMG signals were recorded throughout the experiment. B) The arrival of the stimulus triggered a 3 s count, after which a red light (go signal) turned on. The TMS pulses were delivered at 500 ms or 250 ms prior to the go signal.

Participants sat on a comfortable chair in front of a table where the stimulus (cylinder containing the emotional laden object) was presented on a sliding slab by an experimenter sitting behind a black curtain. They did not see the experimenter at any time. At the beginning of each trial, the left arm of the participant was positioned with the palm facing down over the table (initial position) (Figure 1A). Three seconds after each stimulus presentation, a red light positioned in front of the stimulus was turned on (go signal). Immediately following this signal, the participant picked up the stimulus with his left hand, brought it close to his chest, put it down on the sliding slab and returned his hand to the initial position (ACTION condition). The task was performed with the left arm, based on previous data showing that the effects on motor preparation are more evident for the non-dominant limb [45]. In a second experimental condition, participants were instructed to observe the stimuli and to not move after the red light was turned on (NO-ACTION condition). The right hand was placed on a pillow under the table throughout the experimental session in both conditions. The time interval that elapsed between the presentation of the stimulus and the go signal was considered the preparatory period. Figure 1B presents the sequence of the experimental procedure.

A training session was used to familiarize the participants with the setup, and the experiment was started when the participant reported being comfortable with the task. Three stimuli (one from each emotional category) not employed in the experimental session were presented as described above for training.

The experimental session consisted in four blocks. Two of them were ACTION and the other two, NO-ACTION condition. There were 42 trials per condition (14 unpleasant, 14 neutral, and 14 pleasant).Thus the total number of trials per valence was 56 per participant. TMS pulses were delivered randomly at 500 ms (in half of the trials) and at 250 ms (in the other half) before turning on the red light (go signal). Each emotional stimulus was presented randomly only once in each block. Thus, there was no repetitive exposition to the emotional stimuli throughout the experiment. This caution was taken since the affective response can change over time [46]. Blocks were presented in a pseudo-randomized order. Half of the participants performed the task beginning with the ACTION block followed by the NO-ACTION block, whereas the other half performed the task beginning with the NO-ACTION block followed by the ACTION block. Blocks were separated by approximately 3 min of rest. During this period, instructions concerning the upcoming block were repeated.

### Stimulus Rating

The interaction with each of the 42 stimuli during the TMS session was evaluated at the end of the experiment in valence and arousal dimensions. Upon stimulus presentation, participants had 10 s to classify how they had felt about their interaction with each stimulus in the affective rating scale (SAM) [44] using the same procedure described in the stimulus selection session. Ratings were then averaged per participant and the total duration of the experiment was approximately 90 min.

### Transcranial Magnetic Stimulation (TMS)

For TMS pulse application, a figure-of-eight coil powered by a MagPro stimulator (MagVenture, Denmark) was employed. A cap containing a 1 cm^2^ spaced grid was positioned over the participant's skull to guide the TMS coil placement. Earplugs were provided to protect the participant's hearing. The coil was positioned tangentially over the optimal scalp location of the right primary motor cortex with the handle pointing downwards. First, the optimal position (hot spot) for eliciting MEPs from the *first dorsal interosseous* (FDI) muscle was identified. The resting motor threshold (rMT) was then defined as the minimal intensity needed to evoke MEPs larger than 100 µV peak-to-peak amplitude in this muscle in at least three of six pulses. The stimulation intensity was then set at 120% of the FDI motor threshold to evoke MEPs in the FDI muscle.

### Electromyographic Signal Acquisition

The electromyographic (EMG) signal was recorded using two pairs of Ag-AgCl electrodes, arranged in a bipolar montage over the belly of the FDI and *extensor digitorum communis* (EDC) muscles. MEPs elicited by the TMS pulses were recorded from the FDI. Given that the EDC acts as a wrist stabilizer and is recruited early in reaching and grasping movements, its activity was used as a marker of movement onset to measure the response time in the ACTION condition. Herein, the response time was therefore defined as the time interval elapsed between the go signal and the moment at which the EMG activity of the EDC reached 5% of its maximum amplitude (Figure 1B). EMG activity was recorded using an EMG100 acquisition module coupled to an MP150 amplifier (BIOPAC Systems Inc., Goleta, CA, USA) and stored on a computer for offline analysis. Data were sampled at 20 KHz and band-pass filtered between 10 and 5 KHz with a 60 Hz notch filter.

### Data Analysis

MEPs were quantified based on their latency and peak-to-peak amplitude using MATLAB routines (Mathworks, USA). This routine was devised to open the recorded EMG files and to segment the EMG epochs corresponding to each trial. The beginning and the end of each motor evoked potential (MEP) were marked manually on each trial. The latency was computed by counting the time elapsed between the moment of the TMS trigger and the beginning of the MEP response. The MEP amplitude was calculated by measuring the peak-to-peak amplitude. Data was then exported to Microsoft Excel, and the latencies and the MEP amplitude values for specific condition were organized per participant. The root-mean-square (RMS) of the EMG activity 200 ms prior to the TMS pulse was measured to ensure that the EMG baseline activity remained lower than 10 µV for all experimental conditions. Outlier detection was computed by calculating the mean latency and mean MEP amplitude for each specific condition per participant. Latency and MEP amplitude values exceeding two standard deviations from the mean were computed as outliers and discarded. Based on this criterion, less than 20% of the trials were discarded from the analyses. The number of discarded trials did not differ among valence categories [F (2, 26)  = 0.40, p = 0.67].After removing the outliers, MEP amplitudes were normalized by computing a ratio for the unpleasant and pleasant stimuli by reference to the neutral stimuli per trial within block and within condition. Finally, four participants that lost more than 20% of the trials were excluded from all analyses.

Response time values were analyzed using two criteria. First, trials in which participants began the movement before the go signal were computed as an error. Based on this criterion, 5% of the trials were excluded. After that, the values exceeding two standard deviations of the mean were then discarded, and the average for each participant across action blocks was calculated.

### Statistical Analysis

Statistical analysis of all measured parameters was performed using a repeated-measures ANOVA with the SPSS statistical package (SPSS; San Rafael, CA). Tests of normality were performed to determine the probability that the sample came from a normally distributed population (Shapiro-Wilk's W test, p>0.05). Sphericity of the data was verified before each test using Mauchly's test (for all tests: p≥0.05). For all analyses, the level of significance was set to 0.05 unless stated otherwise. Duncan's post-hoc analysis was employed to test individual comparisons whenever a statistical significance was found. The partial eta squared statistics (η_p_^2^) was computed and reported.

## Results

### Stimulus Rating

**Figure 2:**
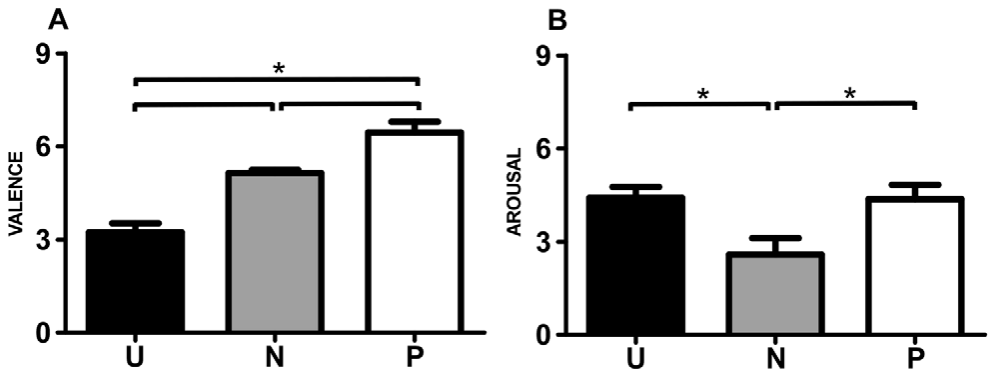
Stimulus ratings collected for the 42 stimuli. A) Scores for the valence dimension. B) Scores for the arousal dimension. U =  unpleasant, N =  neutral and P =  pleasant (*p<0.05).

As expected, the one-way repeated-measures ANOVA yielded a main effect for valence (neutral, pleasant, and unpleasant) [F (2,18)  = 29.97, p<0.01, η_p_^2^ = 0.75]. Post hoc analysis revealed that judging the interaction with the unpleasant stimuli (mean 3.24±0.92 standard deviation) scored significantly lower in valence than that with the neutral (5.14±0.33) and pleasant stimuli (6.44±1.09), whereas the neutral stimuli scored significantly lower than the pleasant stimuli (p<0.01). In addition, there was a main effect for arousal [F (2,18)  = 9.54, p<0.01, η_p_^2^ = 0.52]. Post-hoc analysis revealed that the unpleasant (4.41±1.10) and pleasant stimuli (4.37±1.44) scored similarly in terms of arousal (p = 0.41), both valence stimuli scoring significantly higher than the neutral stimuli (2.59±1.57, p<0.01) (Figure 2).

### Corticospinal excitability (CSE)

**Figure 3:**
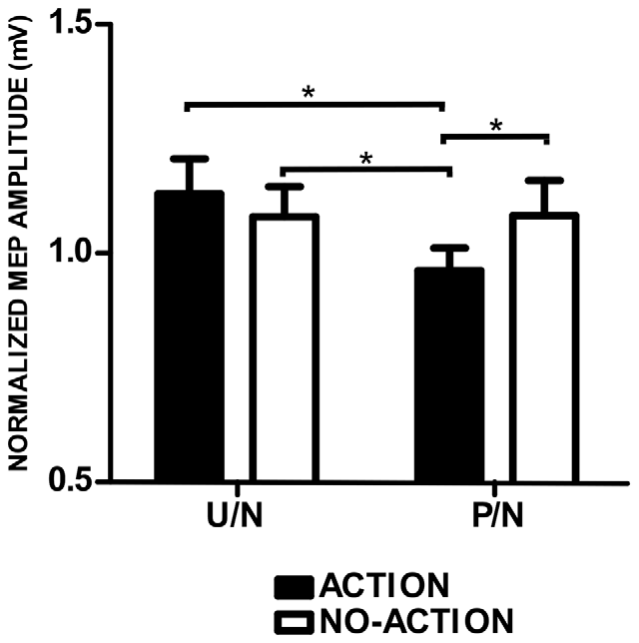
Motor evoked potential amplitude ratios. In the ACTION condition (black bars) the MEP amplitude ratio was higher during the preparation to grasp the unpleasant stimuli compared to the pleasant stimuli. However, there was no valence effect for NO-ACTION condition. Furthermore, the MEP amplitude ratio for the pleasant stimuli in the ACTION condition was smaller than pleasant and unpleasant stimuli ratio in the NO-ACTION condition (white bars). There was no effect for unpleasant stimuli by comparing conditions (ACTION and NO-ACTION). U/N  =  unpleasant/neutral, N =  neutral and P/N  =  pleasant/neutral (*p<0.05).

A three-way repeated-measures ANOVA with condition (ACTION and NO-ACTION), valence (unpleasant/neutral and pleasant/neutral), and stimulation time (250 and 500 ms before movement onset) was conducted to assess differences in the CSE for the FDI muscle. It resulted in a significant condition x valence interaction [F(1, 9)  = 6.72; p = 0.03, η_p_^2^ = 0.43]. Post-hoc analysis revealed that the CSE ratio in the ACTION condition was larger for the unpleasant stimuli (mean 1.13±0.24 standard deviation 0.24) than for the pleasant stimuli (0.96±0.16) (p = 0.008). Moreover, there was no valence effect for the NO-ACTION condition (p = 0.96). Additionally, the CSE ratio for the pleasant stimuli in the ACTION condition was smaller than for the pleasant (1.08±0.24, p = 0.035) and unpleasant (1.08±0.21, p = 0.032) stimuli ratio in the NO-ACTION condition. However, there was no difference between the unpleasant stimuli ratio by comparing ACTION and NO-ACTION conditions (p = 0.33) (Figure 3). Finally, there was no significant effect for condition [F(1, 9)  = 0.63; p = 0.45, η_p_^2^ = 0.07], valence [F(1, 9)  = 3.69; p = 0.09, η_p_^2^ = 0.30] and stimulation time [F(1,9)  = 1.75; p = 0.58, η_p_^2^ = 0.04] nor any other interactions.

### Response Time

A two-way repeated-measures ANOVA yielded no significant effect for time [F (1,9)  = 1.01, p = 0.34, η_p_^2^ = 0.01], valence [F (2,18)  = 0.37, p = 0.70, η_p_^2^ = 0.04] or any interaction [F (2,18)  = 0.22, p = 0.80, η_p_^2^ = 0.02] in response time.

## Discussion

In this study we examined the effects of grasping emotion-laden stimuli on CSE by applying TMS pulses in the motor cortex during motor preparation. Participants grasped stimuli with neutral, pleasant, or unpleasant valence in the ACTION condition and only observed the same stimuli without acting on them in the NO-ACTION condition. Stimuli ratings for the unpleasant and pleasant stimuli were similar in terms of arousal but differed in terms of valence. The CSE ratio preceding the grasping movement was lower for the pleasant stimuli and higher for the unpleasant stimuli during the ACTION condition. Moreover, the CSE ratio for the pleasant stimuli was significantly lower in the ACTION condition compared to the NO-ACTION condition. No difference was found in CSE ratio between the unpleasant stimuli by comparing ACTION and NO-ACTION conditions. Finally, no valence effect was found for the NO-ACTION condition.

Motor preparation involves a change in the expression of a state of the organism that relates to the execution of a forthcoming movement. Recent TMS studies have shown that this change endows a large suppression of CSE during motor preparation that could reflect the recruitment of inhibitory cortical and/or spinal circuitry [47]–[58]. This suppression was described as being crucial to prevent a premature initiation of a planned response [49], [50], [57]. Duque and Ivry (2009) measured the CSE during movement preparation as participants performed a bimanual force choice-reaction time task. They showed that when the hand movement was cued, the CSE was more suppressed for the cued hand compared to the uncued hand. This effect has been interpreted as reflecting an “impulse-control mechanism” to avoid a premature response in the execution of the intended action, thus ensuring the precise movement timing.

A similar impulse control mechanism may be at play when an individual prepares to act upon emotion-laden stimuli. Herein the CSE ratio preceding the grasping of the pleasant stimuli was lower than for the unpleasant stimuli. Likewise, lower readiness potential amplitude was found in motor-related areas preceding the grasping of pleasant stimuli [18]. This was taken as evidence that preparing to grasp pleasant objects triggered approach-like motor repertoires congruent to the action of grasping. Additionally, CSE for pleasant stimuli was lower during the preparation to grasp the object compared to the condition when the participants were oriented not to move after the go signal (NO ACTION condition). It can be hypothesized that the pleasant stimuli triggered an urge to move that required greater suppression, reflecting an enhanced impulse control effect preceding actions towards pleasant stimuli. Accordingly, Albert et al. (2010) oriented participants to move (Go trials) or not (Nogo trials) whereas emotional pictures were presented and showed that withholding an imminent response (Nogo trials) in positive contexts required more inhibitory control than in negative contexts [19], [20]. To our knowledge, this is the first report of a reduced CSE excitability preceding the grasping of pleasant stimuli.

Although there was a higher CSE ratio for unpleasant as compared to pleasant stimuli during motor preparation, no difference was found for the unpleasant stimuli ratio when ACTION and NO-ACTION condition were compared. It could be that CSE modulation tends to go in the same direction both preceding the grasping of unpleasant objects as well as during their observation. In both situations, the unpleasant stimuli might have activated withdrawal networks in the brain and/or the corticospinal pathway. Accordingly, there is evidence that increased activity in motor-related areas both when a simple reaction time task was performed after viewing unpleasant pictures [17] and preceding the grasping of unpleasant stimuli [18]. Likewise, larger CSE excitability has been consistently described to occur during the mere observation of unpleasant pictures [23], [26], [27].

Previous TMS studies have shown arousal modulation on CSE driven by affective picture viewing preceding movement execution [24], [28]. Coombes et al. (2009) measured CSE before the extension of wrist and fingers during the presentation of emotional pictures. They did not find any difference between pleasant and unpleasant conditions, although a significant effect between unpleasant compared to neutral condition was found when CSE was measured immediately before the task. In van Loon et al. (2010), the participants were instructed to press a button to indicate if two symbols were equal or different while viewing task-irrelevant pleasant, neutral, or unpleasant pictures. The results showed that the CSE was greater while viewing the unpleasant and pleasant pictures compared to the neutral pictures preceding the task execution, except for the moment closest in time to the imminent response, when the CSE became greater for the unpleasant condition than for the neutral and pleasant conditions.

Although the emotional effect on CSE described by these studies corroborate with higher CSE ratio for unpleasant stimuli preceding the grasping movement, there are methodological differences between the present study and those studies that should be considered. Firstly, those TMS results did not explore the CSE when participants were effectively planning the movement. In one case, TMS pulses were applied simultaneously with the go signal [24] and, in the other one, TMS pulses were delivered between the presentation of the imperative (go signal) and the response issuing [28]. Thus, MEPs were obtained during the movement onset and not during the movement preparation. It has already been demonstrated that TMS effects on motor preparation can diverge significantly depending on the time of pulse delivery [47], [50]. In contrast, we chose to investigate emotional effects upon CSE at 500 and 250 ms before movement onset. Indeed, the effects of emotional picture viewing upon CSE described in the previous studies seem more consistent with a modulation based on arousal rather than valence. A possible explanation for those results could reside in the fact that the source of the emotion did not match the action's goal. In the current study we tested specifically if the action of grasping a valence-laden object would affect CSE during the preparatory period and we found evidence in favor of a CSE modulation by the valence of the stimulus.

Regarding the emotional influence on response time, unpleasant stimuli have been shown to slow down the response in simple reaction time paradigms [9], [12], [19], [59]. However, faster reaction times were recorded for unpleasant pictures compared to pleasant and neutral pictures when the participants were instructed to make extension movements [11], for grasping movements in a specific disgust context [14], or for the saccadic reaction time in a fear context [15]. In contrast, Coombes et al. (2012) showed that there was no effect of the emotional category (unpleasant, pleasant, and neutral) on the reaction time of a precision grip force task. Contradictory effects of emotion on reaction time have been shown with TMS paradigms as well. For example, Coombes et al. (2009) reported that participants were faster at wrist and finger extension when exposed to unpleasant pictures compared to pleasant and neutral pictures. However, no effect of emotional picture viewing was reported on a choice-reaction time task [28]. Likewise, we found a lack of emotional modulation upon response times. Category-specific effects may explain differences among these studies. Calvo and Avero (2009), for example, showed that the reaction time differed for scene categories, although they were equivalent in valence and arousal [60].

In conclusion, we have shown that motor preparation is affected by the emotional valence of the stimulus to be grasped. Specifically, we report a divergent effect where the pleasant stimuli decreased and the unpleasant stimuli increased the CSE during motor preparation. This dissociation may reflect the recruitment of networks throughout the corticospinal pathway that are involved, respectively, in the readiness or the unwillingness to act.
